# Development and Validation of a Questionnaire to Assess Multimorbidity in Primary Care: An Indian Experience

**DOI:** 10.1155/2016/6582487

**Published:** 2016-02-07

**Authors:** Sanghamitra Pati, Mohammad Akhtar Hussain, Subhashisa Swain, Chris Salisbury, Job F. M. Metsemakers, J. André Knottnerus, Marjan van den Akker

**Affiliations:** ^1^Indian Institute of Public Health, Bhubaneswar, Public Health Foundation of India, Bhubaneswar, Odisha 751024, India; ^2^Division of Epidemiology and Biostatistics, School of Public Health, The University of Queensland, Brisbane, QLD 4006, Australia; ^3^Centre for Academic Primary Care, School of Social and Community Medicine, University of Bristol, Bristol BS8 2PS, UK; ^4^Department of Family Medicine, School Caphri, Maastricht University, 6200 MD Maastricht, Netherlands; ^5^Department of General Practice, KU Leuven, 3000 Leuven, Belgium

## Abstract

Multimorbidity remains an underexplored domain in Indian primary care. We undertook a study to assess the prevalence, correlates, and outcomes of multimorbidity in primary care settings in India. This paper describes the process of development and validation of our data collection tool “Multimorbidity Assessment Questionnaire for Primary Care (MAQ-PC).” An iterative process comprising desk review, chart review, and expert consultations was undertaken to generate the questionnaire. The MAQ-PC contained items on chronic conditions, health care utilization, health related quality of life, disease severity, and sociodemographics. It was first tested with twelve adults for comprehensibility followed by test-retest reliability with 103 patients from four primary care practices. For interrater reliability, two interviewers separately administered the questionnaire to sixteen patients. MAQ-PC displayed strong internal consistency (Cronbach's alpha: 0.69), interrater reliability (Cohen's Kappa: 0.78–1), and test-retest reliability (ICC: 0.970–0.741). Substantial concordance between self-report and physician diagnosis (Scott Kappa: 0.59–1.0) was observed for listed chronic conditions indicating strong concurrent validity. Nearly 54% had one chronic condition and 23.3% had multimorbidity. Our findings demonstrate MAQ-PC to be a valid and reliable measure of multimorbidity in primary care practice and suggest its potential utility in multimorbidity research in India.

## 1. Introduction

Multimorbidity, the concurrent presence of two or more chronic conditions in individuals, is emerging as a daunting health challenge globally with substantial impact on health care utilization, quality of life, and health outcomes [1, 2]. Furthermore, low and middle income countries (LMICs) with socioeconomic development and westernization of lifestyle are no longer immune to this challenge as demonstrated by the reported high prevalence of multimorbidity in Brazil, Ghana, Indonesia, and Vietnam [3–5]. Similar to other LMICs, India, home to one-fourth of the world's population, is exhibiting a rising trend of chronic diseases and thus multimorbidity could be an attendant phenomenon [6–8]. The sheer volume of India's population with concomitant magnitude of multimorbidity can place critical demands on existing health care delivery systems [8]. Contrastingly, multimorbidity is still underexplored in India with the available evidence being mostly from secondary data sources, confined to selected population groups and encompassing few chronic conditions [9]. This may not be representative of the real magnitude since measurement methods strongly influence the observed prevalence of multimorbidity thus underscoring the need for an explicit, validated measurement tool [10].

Our systematic review of multimorbidity studies in the south of Asia has confirmed the lack of uniformity in assessment of multimorbidity with the conspicuous absence of reports from primary care in India [9]. This is a vitally important knowledge gap, as primary care constitutes the scaffold of health care delivery in the country and the complex care needs of multimorbid patients require appropriate redesigning of primary care services [11]. Moreover, only prevalence of multimorbidity may not be sufficient to inform health services as the typology of conditions and severity level also influence the health care to be delivered and the subsequent outcomes [11]. Although clinical data retrieved from patients' records can yield accurate estimation of multimorbidity, our chart review of four urban primary care practices found that multiple chronic conditions are often not recorded in practice. Furthermore, unlike western countries, primary care databases are not routinely maintained in India; hence extraction of medical records from specialist facilities will present a skewed picture [12].

Aiming at addressing the aforementioned knowledge gap, we undertook a study to explore the magnitude of multimorbidity and its correlates and outcomes in a primary care setting.

It is expected that this information would help public health researchers in India and similar settings to estimate the magnitude and impact of multimorbidity in primary care practice populations.

## 2. Design and Methods

The study was undertaken in Odisha, an Indian state (approximate population share of 4% of the total population of India) with average health indicators and comparable health system characteristics [13]. Considering the absence of standardized assessment instruments, with proper medical records being unavailable, we first developed and contextualized a tool so as to identify and quantify multimorbidity. We decided to use patient self-reports to elicit information, as they have demonstrated predictive ability of real morbidity [14, 15].

**Figure 1:**
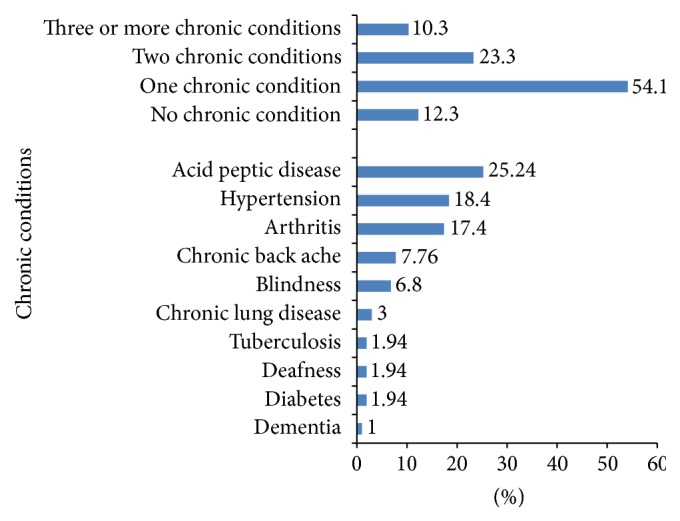
Prevalence of chronic conditions.

We aimed to develop and validate our Multimorbidity Assessment Questionnaire for Primary Care (MAQ-PC). To examine multimorbidity in primary care in Indian context, with no gold standard available, we followed an iterative process to design a comprehensive tool. This comprised two phases. The first phase is the development of the questionnaire, selecting the domains and their measurements, translating the questionnaire to local language for cultural adaptability, and testing its comprehensibility. The second phase involved reliability and validity testing. The steps are outlined in Figure  1 (supplementary file in Supplementary Material available online at http://dx.doi.org/10.1155/2016/6582487).

### 2.1. Selection and Development of Domains

Following domains were selected by the research team through literature review and consultation with an expert group and six primary care physicians. The expert group comprised two senior faculty members of the Department of Family and Community Medicine at the state medical colleges, two clinicians from the Odisha branch of the Indian Medical Association (IMA), two diseases control program managers from the state public health directorate, and four internationally acclaimed researchers in multimorbidity. It was decided to select six primary care physicians working in public and private settings. The three private primary care facilities were selected in consultation with the Odisha branch of IMA, while for public primary care facility selection the state public health department's advice was sought. To ensure representativeness, one public facility and one private facility each from the rural, urban, and tribal regions were selected. 

*(1) Multimorbidity Estimation*. To measure multimorbidity, we decided to have an exhaustive list of chronic diseases commonly prevalent in primary care. We first undertook a systematic search of the available studies in India and other south Asian countries to determine if any of them used a list for the most frequently reported chronic conditions [9]. Next, chart review of four primary care practices (two each from urban and rural area) was done to add relevant chronic conditions to the list generated from systematic search. The draft list was shared with the six primary care physicians who were requested to indicate how important (marginal or very severe) they considered each particular chronic disease and to mention additional diseases to the list, if any. Finally, a consolidated list of 18 conditions (Table 1) was incorporated in the questionnaire. To ascertain the presence of chronic conditions, we used patient self-report [15]. The questions were phrased to elicit whether the patient had ever been told by a doctor or any other health care provider that they had any of the listed chronic health problems. We used simple vernacular language (Odiya) that could be understood by individuals without any prior medical knowledge (Have you even been diagnosed by a physician with…?). In addition to the self-report, we used the Patient Health Questionnaire-9 to capture undiagnosed depression [16]. 

*(2) Outcomes.* To explore the impact of multimorbidity, we included self-reported severity, health related quality of life, and health care utilization. We did not include health care expenditure for the sake of brevity:

 
*(a) Severity Assessment.* Functional limitation was used as proxy for disease severity. For each identified morbidity, we included a subquestion asking how much the particular health problem gets in the way of daily activities (e.g., not at all, a little, or a great deal) [17].
 
*(b) Health Related Quality of Life (HRQL).* To explore health related quality of life, we included two questions on self-rated physical and mental health (e.g., poor, good, or excellent) and SF-12 (already validated for Indian population) [18].
 
*(c) Health Care Utilization.* To examine the health care utilization, we included questions that asked about number of outpatient consultations and inpatient admissions at different health care facilities in the past twelve months and medication use for each reported chronic illness [19].

*(3) Covariates.* We included age (in completed years), sex (male/female), place of residence (urban/semiurban/rural), ethnicity (social caste/tribe), religion (Hinduism/Islam/Christianity/others), educational level (illiterate/primary education/high school or secondary education/graduate and above), marital status (never married/currently married/separated or divorced/widow or widower), and annual family income [13]. 

We hypothesized that the MAQ-PC would identify patients to have multimorbidity when they have self-reported multiple chronic conditions; we expected that the overall judgment of self-reported measures of multimorbidity would correlate strongly with physician diagnoses and would also have high internal consistency with other domains (outcomes).

### 2.2. Translation and Cultural Adaptation

We followed a standard process to ensure the quality of translation (Figure  2, supplementary file). Primary forward translation from international English into vernacular language (Odiya) was performed by two translators independently according to the standard WHO protocol [20]. The primary translation was then evaluated for authenticity by two primary care physicians well versed in both languages. The primary translators discussed apparent differences between the translated versions with the research team and then agreement was reached.

### 2.3. Expert Consultation, Cognitive Debriefing, and Pretesting

The primary care physicians and international experts were consulted to respond to the questionnaire to obtain an initial impression of how easy the questions were to read out, understand, and answer and their feedback was incorporated. Next, the instrument was cognitively tested with 12 adults of diverse ages and socioeconomic strata (six men and six women) for comprehensibility. Structured interviews were performed with them to evaluate whether all the items in the MAQ-PC were understood as intended and to examine the appropriateness of the questionnaire in the local context. The responses were evaluated by the research team and the translation team to check if required information is being captured or not. Based upon it, the questionnaire was revised. Next step involved a small scale operational testing of the questionnaire in one primary health centre to check the logistic feasibility. The time taken to complete the questionnaire was around 20–25 minutes.

Based on the cumulative observations of above three processes, we incorporated few changes in the MAQ-PC. We added open options for three additional chronic conditions not enlisted in our questionnaire. Insurance availability and utilization were added. Since we found difficulty in capturing near exact information for income, we included an additional measure of socioeconomic status, above poverty line (APL)/below poverty line (BPL), adopted by the state government for categorizing people based on income [21]. As the patients expressed difficulty in recalling the year of diagnosis and chronology of appearance for each chronic condition, these questions were omitted. An interviewer's manual was prepared detailing out the instructions for each question. The final version of MAQ-PC is described in Table 2.

### 2.4. Piloting

We examined the reliability and validity of MAQ-PC final version through a large scale pilot testing in four (two public and two private) purposively selected primary care practices in different cities and regions (rural, urban, semiurban, and tribal) in the state. Adult patients over 18 years of age attending outpatient clinic of these primary health care centres were included as study participants. Exit interview was conducted with eligible patients soon after their physician consultation. Informed consent to take part in the interview was obtained from each patient after briefing them about the study and its objectives. A total of 120 patients were recruited through a systematic random sampling from the selected four facilities. Four specially trained nurses administered the questionnaire to patients and examined the physician's prescription. The data collection took place under the direct supervision of the principal investigator (SP) and the research team.

All 120 patients were then invited to take part in the two-day retest. As there was increased likelihood of getting different responses to the question “disease severity and activity limitation” because of the treatment or medication, we confined our retest analyses to day 2. A total of 103 participants turned up for the retest and were then administered the MAQ-PC by the same nurses. For each reported chronic condition, we examined physicians' prescriptions and noted the diagnoses. Additionally, to test interrater or interobserver reliability, another 16 patients were purposively selected and MAQ-PC was administered to them by two members of the research team (MAH and SS) within 24 hours. Each observer was blinded to the results of the other assessment. The agreement was checked by the principal investigator (SP).

All data were entered and analysed using Statistical Package for the Social Sciences version 20 (SPSS Inc., Chicago, IL). Descriptive statistics were calculated and presented as proportion, mean, and standard deviation (SD). The prevalence of multimorbidity was measured in terms of the presence of two or more self-reported chronic conditions. The mean score, interclass correlation coefficient, and Cronbach's alpha coefficient for each domain were calculated to examine the internal consistency using the Kuder-Richardson formula [22]. For interobserver reliability, we determined the observed agreement between two interviewers using Cohen's Kappa statistics [23]. The mean score for each domain was computed to estimate the Kappa value. The concurrent validity of MAQ-PC was assessed by testing the hypotheses that MAQ-PC self-reported morbidity correlates strongly with diagnosed multimorbidity. The level of concordance (self-reports and physician's prescription) for each condition was calculated using Scott Kappa statistics (prevalence-adjusted bias-adjusted Kappa).

## 3. Results

### 3.1. Sample Characteristics

To assess if our study sample was representative of the primary care population, we studied key characteristics of included patients (Table  3, supplementary file). Out of 103 respondents who participated in test and retest (86% of first sample), 45% (*n* = 46) were female. The mean age of the study participants was 44.96 ± 5. 32 years with no significant sex difference (female, 45.9, versus male, 44.2).

### 3.2. Multimorbidity

Nearly 54% of respondents had at least one self-reported chronic condition enlisted. The prevalence of multimorbidity was 23% (male, 22%, versus female, 25%) and around 10% of respondents had three or more chronic conditions. Frequently reported chronic conditions were acid peptic disease (25%), arthritis (17%), hypertension (18%), and chronic back pain (8%), while stroke, cancer, renal disease, and depression were reported very less (Figure 1).

### 3.3. Internal Consistency

The overall consistency of the MAQ-PC was found to be 0.69 for all 52 items with Cronbach's alpha value for individual domain ranging from 0.66 for health related quality of life to 0.89 for depression (Table 3).

### 3.4. Interobserver Reliability

Both observers reported similar prevalence of multimorbidity. We observed a substantial to almost perfect agreement between the two interviewers. Lowest agreement was seen for depression (Table 4).

### 3.5. Test-Retest Reliability

The test-retest reliability score for each domain is denoted in Table 5. We found strong test-retest correlation in multimorbidity assessment domain [ICC: 0.970], followed by quality of life physical component score [ICC: 0.912] and disease severity [ICC: 0.903]. Lowest correlation was seen for the item self-rated overall health [ICC: 0.741].

### 3.6. Concurrent Validity

The correlations between the self-report and physician's prescription are presented in Table 6. The summative multimorbidity score between the first and follow-up interviews was strongly correlated thus demonstrating self-report to be adequately predictive of diagnosed morbidity. The level of agreement was highest for visual problem, tuberculosis, and dementia while being moderate for diabetes and hearing problems.

### 3.7. Ethical Consideration

The study was conducted in accordance with the Declaration of Helsinki. It was approved by the Institutional Ethics Committee of Public Health Foundation of India, New Delhi, and necessary permission was granted by the Government of Odisha. Written informed consent was obtained from all respondents following an explanation of the study's aims and procedures. Participation was purely voluntary and all steps have been taken to ensure confidentiality.

## 4. Discussion

Information on presence and composition of multimorbidity could inform routine clinical practice and impetus for research. Since the magnitude of multimorbidity is largely reliant upon the way it is measured, we designed a comprehensive tool, MAQ-PC, to elicit data on self-reported prevalence, correlates, and outcomes of multimorbidity in patients attending primary care practices [24]. The questionnaire intended to measure individuals' count of chronic conditions, outcomes (severity, self-rated health, quality of life, physician consultation, and medications), and sociodemographic correlates. We found multimorbidity prevalence to be higher than previously reported findings [24]. This is expected, as we included a larger number of chronic conditions and collected data from patients attending primary care facility.

In this pilot, the MAQ-PC identified hypertension, arthritis, and acid peptic disease as the most common morbidities, while stroke, cancer, renal disease, and depression were the least frequently mentioned morbidities. As health system characteristics influence the type of conditions patients would present with, the conditions which were more frequent could be predominantly diagnosed and treated in primary care [25]. The extreme low number of morbidities, stroke, cancer, depression, and renal disease, could be due to the low prevalence of these conditions in the community and a small sample size of our pilot [26]. Moreover, some of these patients might be consulting specialists for their illnesses, which could be another contributing reason [25]. Interestingly, even though depression was underreported, a good proportion of undiagnosed patients had higher PHQ-9 score. This suggests that these patients either have not attributed much significance to related symptoms or may not have consulted the physician at all.

We observed the MAQ-PC to exhibit significant test-retest reliability with a substantial degree of agreement between self-reported chronic condition and physician diagnosis (derived from prescription and medicine verifications). Such high level of agreement between the self-reported and physician diagnoses suggests the utility of the patient's self-report as a valid proxy measure for these conditions. For few conditions, where the agreement was relatively lower, the patients might be having the disease in milder form or initial stages and can perceive the symptoms though not being detected by the treating physicians. Another plausible explanation could be the fact that patients are not fully aware of their prevailing illness despite having confirmed diagnosis. The latter might be related to the lower health literacy as majority of our patients had lower literacy [27]. Further analysis into the predictors of concordance might yield useful insights.

### 4.1. Strengths and Limitations

Instruments contingent on availability and accuracy of medical records may have limited utility for clinical and research purposes owing to the deficient routine data management system in resource-limited countries like India [28]. Given the understanding that primary care practice characteristics in LMICs may not be comparable with those of western countries, this work for the first time has developed a multimorbidity assessment tool and contextualized it for Indian primary care.

When compared to multimorbidity measurement methods available till date in LMICs, our approach and instrument are scientifically superior in many aspects. The questionnaire was generated through an iterative process of desk review and chart review, translation, and cultural adaptation, pretested with cognitive interviews including negotiation between the primary care physicians and the research team. These steps helped assure content and face validity. This is reflected by the questionnaire displaying good psychometric properties with Cronbach's alpha and ICC indicating it to be internally consistent and reliable in this setting. Furthermore, many of the domains draw on already validated questionnaire which reinforces the robustness.

Our MAQ-PC has positive features of being brief and easily understandable by patients and at the same time being comprehensive enough to include commonly prevalent chronic conditions in primary care patients. Each questionnaire on an average took 20–25 minutes to complete and thus can easily be administered at outpatient setting either by a physician or by other health care professionals. Employing self-report allows identifying multimorbidity by simple count and the results from the item scales can be easily scored and readily interpretable. Moreover, the questionnaire enquires about the treatment and limitations imposed by specific diseases which can be used as a surrogate marker of the severity of the disease.

However, some limitations need to be acknowledged while using this MAQ-PC. It has been shown previously that list of diseases reported on the basis of prescriptions may not be fully accurate, as many conditions remain undiagnosed, so using this method as the gold standard may not be ideal. Additionally, with any questionnaire-based technique, there is a potential for recall bias. Though patients had the option of mentioning any additional diseases that were not listed, it is possible that patients may not recall milder forms of existing comorbid diseases and this may inadvertently leave out some important conditions. We did not elicit information on the duration and order of appearance of individual diseases, thus weakening our severity score. Our outcome assessment is not comprehensive as it did not include health care expenditure as we were apprehensive of time constraint and also our primary objective was to examine multimorbidity prevalence, pattern, and health outcomes. Lastly, we have only examined the appropriateness of questionnaire in primary care patients, thus restricting the possibility of extrapolating to other groups of patients like those attending more specialized care and having complex patterns of multimorbidity. Despite these limitations, we believe that MAQ-PC, being a reliable and valid descriptor of individual chronic morbidities, has utility as a tool for identifying and quantifying multimorbidity in primary care.

### 4.2. Future Research Directions

Future studies need to examine the suitability of MAQ-PC to measure multimorbidity in other outpatient care settings, where medical records are unavailable. Further development of this questionnaire might include specific enquiry about the duration and chronological order of multiple chronic conditions and health care expenditure. Since the number of chronic diseases increases with age and multimorbidity is a frequently observed geriatric phenomenon, it is necessary to test the applicability of this tool in geriatric population particularly.

## 5. Conclusion

To summarize, MAQ-PC is a comprehensive tool for obtaining data on patient self-reported multimorbidity in primary care. Our results demonstrate this questionnaire to be a valid and reliable measure of multimorbidity in a variety of chronic conditions and primary care patients. The instrument also provides information on severity of the individual conditions and impact on quality of life which suits the need in primary care to identify patient groups that might benefit from more coordinated and holistic care. We believe MAQ-PC may find applicability in assessing multimorbidity and its impact, following multimorbidity trajectory, designing therapeutic targets across wide range of health care settings in India.

## Supplementary Material

The supplementary file being provided contains two figures and one table. Figure 1 describes the outline of the MAQ-PC development and validation process. Figure 2 depicts the methods adopted for forward and backward translation of the tool. Table 1 represents the summary statistics of socio demographic variables of the pilot study participants.

## Figures and Tables

**Table 1:** List of chronic diseases.

Diseases included		
Sl. number	Name	Questions asked for self-reported doctor diagnosis
(1)	Diabetes	Yes
(2)	Hypertension	Yes
(3)	Arthritis	Yes
(4)	Acid peptic disease	Yes
(5)	Asthma	Yes
(6)	Heart disease	Yes
(7)	Stroke	Yes
(8)	Chronic kidney disease	Yes
(9)	Chronic liver disease (alcohol)	Yes
(10)	Chronic back ache	Yes
(11)	Tuberculosis	Yes
(12)	Filariasis	Yes
(13)	Visual difficulty	Yes
(14)	Deafness	Yes
(15)	Cancer	Yes
(16)	Dementia	Yes
(17)	Epilepsy	Yes
(18)	Thyroid	Yes

Depression was screened by using PHQ-9.

**Table 2:** Domains, items, and measurement tools in MAQ-PC.

Domain	Measure	Validation process
Chronic conditions		
Diseases (18 with 3 additional open options)	Close ended question of self-reported doctor diagnosed diseases, symptoms, and prescription check	Scott Kappa value
Depression	Patient Health Questionnaire-9	Test-retest reliability and interrater reliability Internal consistency
Medication	Close ended question according to expert group	Test-retest reliability and interrater reliability Internal consistency

Health care utilization		
Frequency of hospital visits in last one year for any chronic disease	Open ended question for outpatient visit in last one year [WHO-SAGE]	Test-retest reliability and interrater reliability Internal consistency
Frequency of inpatient admission in last one year for any chronic disease	Open ended question for hospitalization [WHO-SAGE]	Test-retest reliability and interrater reliability Internal consistency
Number of medicines being taken daily	Open ended question for number of medicines taken	Test-retest reliability and interrater reliability Internal consistency

Health related quality of life		
Self-rated overall health	Scales	Cognitive briefing Test-retest reliability and interrater reliability Internal consistency
SF-12	Mental components Physical components	Cognitive briefing Test-retest reliability and interrater reliability Internal consistency

Severity of the disease		
Limitation in activities due to health problems	Impact of individual chronic disease on activity limitation	Test-retest reliability and interrater reliability
Frequency of hospital visits for current disease	Adopted from WHO-SAGE 2010	Test-retest reliability and interrater reliability

Sociodemographic		
Age of the patient	Annual Health Survey, India	Internal consistency
Gender	Annual Health Survey, India	Internal consistency
Marital status	Annual Health Survey, India	Internal consistency
Education	Annual Health Survey, India	Internal consistency
Net household income per month	Annual Health Survey, India	Internal consistency
Socioeconomic status	According to the government of Odisha	Internal consistency
Religion and social caste	Annual Health Survey, India	Internal consistency
Health insurance	Close ended questionnaire developed	Internal consistency

**Table 3:** Measure of internal consistency of MAQ-PC.

Domains	Number of items	Cronbach's alpha coefficient
Sociodemographic	8	0.741
Health care utilization	3	0.651
Chronic diseases	18	0.712
Depression	9	0.891
Disease severity	2	0.671
Health related quality of life	12	0.664

Overall	52	0.693

**Table 4:** Interobserver reliability (Cohen's Kappa statistics).

Theoretical construct and facets	Observer 1 Mean [SD]	Observer 2 Mean [SD]	Kappa	Strength of agreement
Chronic conditions				
Diseases and other health problems	1.61 [0.86]	1.61 [0.86]	1	Nearly perfect agreement
Depression	1.92 [0.88]	1.83 [0.83]	0.784	Moderate agreement

Health care utilization				
Frequency of hospital visits in last one year	0.98 [1.46]	0.97 [1.39]	0.921	Substantial agreement
Frequency of inpatient admission in last one year	0.30 [0.61]	0.35 [0.76]	0.874	Substantial agreement
Number of medicines taken	0.46 [0.79]	0.39 [0.68]	0.851	Substantial agreement

Health related quality of life				
Self-rated overall health	3.53 [0.68]	3.87 [0.75]	0.812	Substantial agreement
SF-12 mental component score	44.25 [9.64]	45.27 [8.67]	0.791	Moderate agreement
SF-12 physical component score	43.57 [4.72]	43.91 [5.13]	0.786	Moderate agreement

Severity				
Limitation in activities due to health problems	7.00 [5.93]	7.12 [6.15]	0.831	Substantial agreement

Multimorbidity				
Multimorbidity (≥2 chronic conditions)%	13.16	13.16	1	<0.001

**Table 5:** Measures of reliability (test-retest reliability) for different domains of MAQ-PC.

Domains	*N*	Test Mean [SD]	Retest Mean [SD]	*P* value of the difference	ICC^*∗*^
Chronic conditions	103	1.61 [0.86]	1.60 [0.82]	0.932	0.970
Depression	103	1.92 [0.88]	1.86 [0.84]	0.617	0.817
Health care utilization					
Frequency of hospital visits in last one year	103	0.98 [1.46]	0.96 [1.42]	0.920	0.822
Frequency of inpatient admission in last one year	103	0.30 [0.61]	0.33 [0.62]	0.726	0.881
Number of medicines taken	103	0.46 [0.79]	0.49 [0.78]	0.784	0.841
Health related quality of life					
Self-rated overall health	103	3.53 [0.68]	3.41 [0.72]	0.220	0.741
SF-12 MCS	103	44.25 [9.64]	43.95 [9.67]	0.823	0.893
SF-12 PCS	103	43.57 [4.72]	43.61 [5.12]	0.953	0.912
Severity of the disease					
Limitation in activities due to health problems	103	7.00 [5.93]	6.89 [5.60]	0.891	0.903
Multimorbidity (%)	103	23.03	23.01	0.897	0.963

^*∗*^Interclass correlation.

**Table 6:** Concordance between self-reported and physician's prescription based chronic conditions.

Items	Number of cases (*n* = 103)	Number of cases according to prescription (*n* = 103)	Scott Kappa	Strength of agreement
*Chronic conditions*				
Arthritis	26	24	0.71	Substantial agreement
Hypertension	21	20	0.73	Substantial agreement
Diabetes	6	7	0.59	Moderate agreement
Chronic lung disease	7	7	0.69	Substantial agreement
Acid peptic disease	33	32	0.66	Substantial agreement
Thyroid problem	0	0		
Heart disease	0	0		
Stroke	0	0		
Visual problem	11	10	0.95	Nearly perfect agreement
Hearing problem	5	5	0.58	Moderate agreement
Chronic back ache	10	10	0.67	Substantial agreement
Tuberculosis	4	4	1.00	Nearly perfect agreement
Epilepsy	0	0		
Chronic kidney disease	0	0		
Dementia	4	3	0.85	Nearly perfect agreement
Filariasis	0	0		
