# Supplementary material for: Development and Validation of a Questionnaire to Assess Multimorbidity in Primary Care: An Indian Experience
Source: Biomed Res Int. 2016 Feb 7;2016:6582487. doi: 10.1155/2016/6582487 (PMC4761379; doi:10.1155/2016/6582487)
Supplement: Supplementary file 1 — The supplementary file being provided contains two figures and one table. Figure 1 describes the outline of the MAQ-PC development and validation process. Figure 2 depicts the methods adopted for forward and backward translation of the tool. Table 1 represents the summary statistics of socio demographic variables of the pilot study participants. [file 6582487.f1.pdf]

Supplementary File

Figure 1: Outline of MAQ PC development and validation

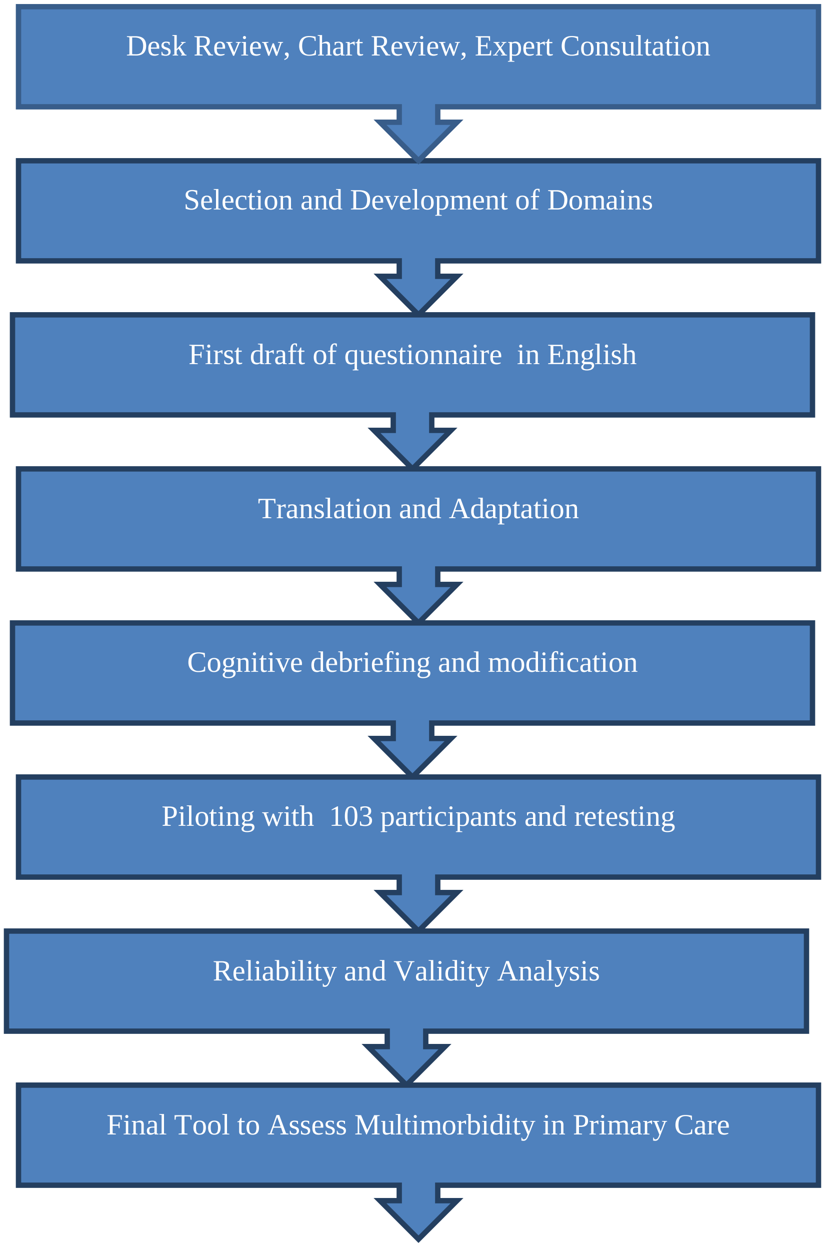

Figure 2: Translation process

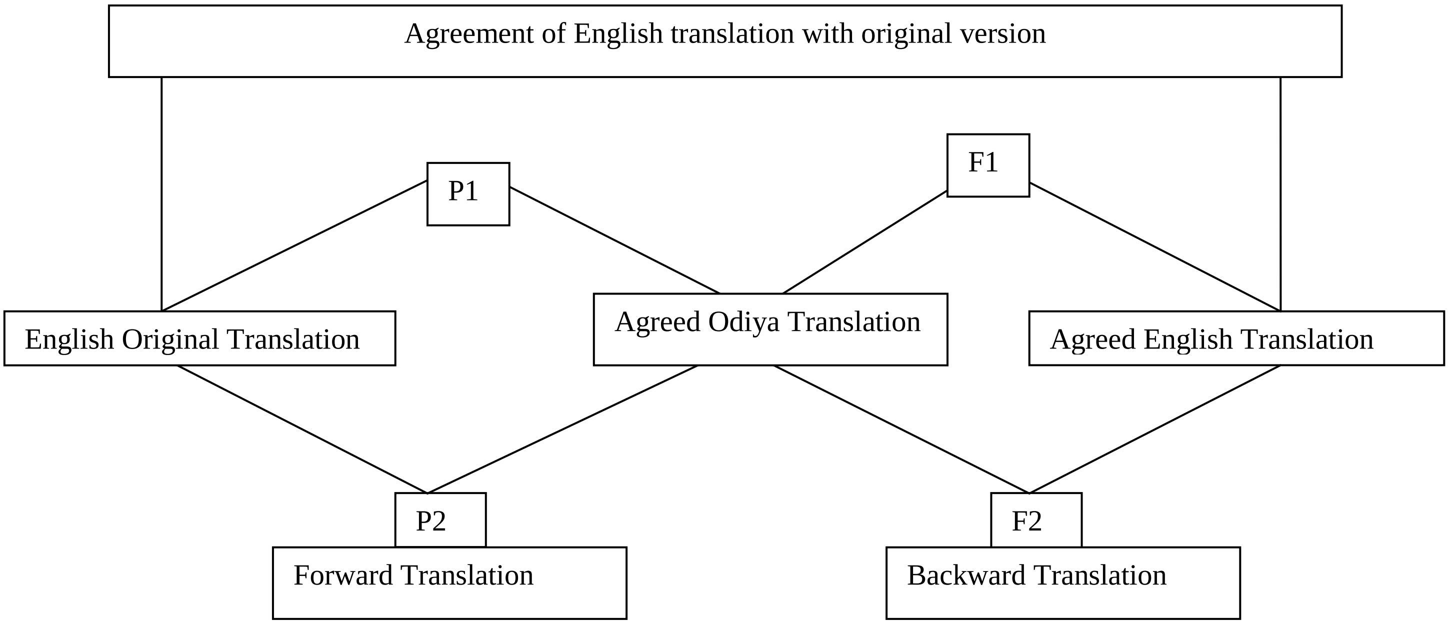

P1/P2: independent translation into Odia

F1/F2: independent translation into English

Table 3: Baseline characteristics of study subjects

Variables	Category	Percentage [95% CI]
Sex	Female	44.66[34.89-54.42]
	Male	55.33[45.57-65.10]
Socio economic status	BPL	61.16[51.59-70.73]
	APL	38.83[29.26-48.40]
Ethnicity	Schedule caste	11.65[05.34-17.95]
	Schedule tribe	39.80[30.19-49.41]
	General	48.54[38.72-58.35]
Age	Male [Mean, SD]	44.19 [39.37-49.01]
	Female [Mean, SD]	45.95[41.18-50.72]
Education	Illiteracy	41.74[32.06-51.43]
	Primary Schooling Completed	33.00[23.77-42.25]
	Secondary/Higher Secondary Schooling Completed	21.35[13.31-29.40]
	Graduation and Above	3.88[0.09-7.67]
